# Pulsed Electromagnetic Fields Improve Tenogenic Commitment of Umbilical Cord-Derived Mesenchymal Stem Cells: A Potential Strategy for Tendon Repair—An In Vitro Study

**DOI:** 10.1155/2018/9048237

**Published:** 2018-07-30

**Authors:** Antonio Marmotti, Giuseppe Maria Peretti, Silvia Mattia, Laura Mangiavini, Laura de Girolamo, Marco Viganò, Stefania Setti, Davide Edoardo Bonasia, Davide Blonna, Enrico Bellato, Giovanni Ferrero, Filippo Castoldi

**Affiliations:** ^1^Department of Orthopaedics and Traumatology, University of Turin, Torino, Italy; ^2^Molecular Biotechnology Center, University of Turin, Torino, Italy; ^3^IRCCS Istituto Ortopedico Galeazzi, Milano, Italy; ^4^Department of Biomedical Sciences for Health, University of Milan, Milano, Italy; ^5^IGEA SpA Clinical Biophysics, Carpi, Modena, Italy

## Abstract

Tendon repair is a challenging procedure in orthopaedics. The use of mesenchymal stem cells (MSCs) and pulsed electromagnetic fields (PEMF) in tendon regeneration is still investigational. In this perspective, MSCs isolated from the human umbilical cord (UC) may represent a possible candidate for tendon tissue engineering. The aim of the study is to evaluate the effect of low-frequency PEMF on tenogenic differentiation of MSCs isolated from the human umbilical cord (UC-MSCs) in vitro. 15 fresh UC samples from women with healthy pregnancies were retrieved at the end of caesarean deliveries. UC samples were manually minced into small fragments (less than 4 mm length) and cultured in MSC expansion medium. Part of the UC-MSCs was subsequently cultured with PEMF and tenogenic growth factors. UC-MSCs were subjected to pulsed electromagnetic fields for 2 h/day, 4 h/day, or 8 h/day. UC-MSCs cultured with FGF-2 and stimulated with PEMF showed a greater production of collagen type I and scleraxis. The prolonged exposure to PEMF was also related to the greatest expression of tenogenic markers. Thus, the exposure to PEMF provides a positive preconditioning biophysical stimulus, which may enhance UC-MSC tenogenic potential.

## 1. Introduction

Tendon repair and regeneration are still an unsolved problem in orthopaedics. Indeed, the tendon healing may be subjected to failure, due to the incomplete restoration of the tendon body and to the lack of osteointegration at the level of the enthesis. As an example, rerupture of the Achilles tendon is frequent with a rate of approximately 5% in the surgically treated cases and up to 12% in the case of conservative treatment [1]. Moreover, surgical reconstructive procedures may obtain good repair of the tendon to the anatomic insertion site, but the integration at the enthesis is often unsuccessful, leading to the formation of a fibrotic scar. Thus, the rerupture rate after rotator cuff repair is approximately 25% of cases and even more in the occurrence of large cuff tears, as shown in literature [2–4]. Thus, an enhancement of the tenogenic process during tendon regeneration and an improvement of the enthesis reconstruction may ameliorate the final results of tendon repair.

Different studies have focused on the key elements of tendon regeneration; in particular, cell preconditioning toward the tenogenic line may represent a possible solution to improve tendon regeneration. Several attempts have been made to increase enthesis and tendon regeneration by adding undifferentiated MSCs with unsatisfying results, as shown by the recent works of Gulotta et al. [5] and Kraus et al. [6]. Conversely, scleraxis gene transduction leads to a satisfactory result in preclinical mouse models of rotator cuff repair [7]. In addition to transgenic techniques, physical forces may exert a positive effect on the cell microenvironment, as observed with extracorporeal shock wave and pulsed electromagnetic fields (PEMF) [8–11]. Thus, a “microenvironmental effect” by biophysical forces may represent an alternative key element to obtain MSC preconditioning toward the tenogenic pathway.

Indeed, PEMF exposure [12, 13] on human tendon determined increased scleraxis and collagen type I expression, as well as increased production of IL-10 and VEGF, also involved in the tendon healing process. These results may suggest a possible “biologic scenario” to improve MSC tenogenic development with the application of PEMF. In this work, we analyzed UC-MCS exposed to PEMF and FGF-2, to investigate the possible role of PEMF exposure in tenogenic differentiation. In a possible future clinical context, the use of an allogeneic source of MSCs is an attractive approach with several advantages, such as the unlimited availability of cells and the absence of patient morbidity. Indeed, the potential wide availability of the umbilical cord after caesarean births allows for the isolation of a considerable number of cells hypothetically available for storage in stem cell factories. Thus, in this perspective, UC-MSCs may be conceived as an “off-the-shelf” ready-to-use cell source to be used for different orthopaedic reconstructive procedures such as tendon repair. Moreover, the umbilical cord may be defined as a “waste material” with intrinsic low ethical concerns for future clinical applications. A promising differentiation potential of UC-MSCs toward mesenchymal cell lines has been recently demonstrated in previous in vitro studies [14–16].

## 2. Materials and Methods

Approvals were obtained both from the Ethical Committee of MBC (Molecular Biotechnology Center), University of Turin, and from the Ethical Committee of Mauriziano Hospital, Turin (Italy) (protocol number: CS792, approved on January 11, 2016).

### 2.1. UC Collection and Processing

After obtaining a specific patient's informed consent, fresh UC samples from 15 women with healthy pregnancies were recovered during caesarean deliveries from the Department of Obstetrics and Gynecology of Mauriziano Hospital (Turin, Italy). UC samples were collected into phosphate-buffered saline (PBS) (Invitrogen, Carlsbad, CA, USA) transfer medium containing 200 mg/100 mL ciprofloxacin (Bayer, Milan, Italy) and 500 IU heparin (Pharmatex, Milan, Italy). Then, cord length and weight were assessed. UC segments were then manually minced into small cuboidal fragments (4–7 mm length). The umbilical cord fragments were seeded in 60 cm^2^ Petri dishes and cultured in expansion medium containing Dulbecco's modified Eagle medium/F-12 (D-MEM) (Invitrogen, Carlsbad, CA, USA), 5% human platelet lysate obtained from healthy donors, 10% fetal bovine serum (FBS), 1X penicillin/streptomycin (Invitrogen, Carlsbad, CA, USA), 1X sodium pyruvate (Invitrogen, Carlsbad, CA, USA), 1X nonessential amino acids (Invitrogen, Carlsbad, CA, USA), and 500 IU heparin (Pharmatex, Milan, Italy).

UC fragments were distributed into different 60 cm^2^ Petri dishes (approximately 40–45 fragments/Petri dish) and incubated in the MSC expansion medium at 37°C in a humidified atmosphere with 5% CO_2_ for up to 2 weeks.

### 2.2. Culture and UC-MSC Immunophenotypic Characterization

Subsequently, UC debris were removed and adherent cells were expanded for 2 additional weeks.

40% of the medium was changed every 3-4 days. Adherent cells (P0) were then trypsinized, centrifuged, resuspended in MSC expansion medium, and replated for one consecutive expansion step at a density of 100–200 cells/cm^2^, until full confluence was reached (P1). Cell confluence at P1 was reached after approximately 14 days (day 42).

At the end of P1, living cells were counted by trypan blue dye.

Immunophenotyping of the expanded UC-MSCs was performed by flow cytometry analysis at P1. The following antibodies were used: CD90-peridinin chlorophyll protein- (PerCP) cyanine dye Cy5.5 (BioLegend, San Diego, CA), CD105-fluorescein isothiocyanate (FITC) (BioLegend, San Diego, CA), CD73-allophycocyanin (APC) (BD Biosciences, San Jose, CA), CD34-phycoerythrin (PE) (BD Biosciences, San Jose, CA), HLA-DR-FITC (BD Biosciences, San Jose, CA), HLA-PerCP (BD Biosciences, San Jose, CA), HLA-ABC-PE, CD29-APC (BD Biosciences, San Jose, CA), CD44-Alexa Fluor (Cell Signaling Technology, Danvers, MA), PE-conjugated anti-mouse immunoglobulin G (IgG) (Southern Biotechnology Associates, Birmingham, Alabama, USA), isotype-matched IgG-FITC (BioLegend, San Diego, CA), IgG-PE (BioLegend, San Diego, CA), and IgG-PE-Cy5 (BioLegend, San Diego, CA) control antibodies. Analysis was performed on a FACScan (Becton Dickinson (BD), Buccinasco, Italy) for at least 10,000 events using CellQuest software (BD, Buccinasco, Italy).

### 2.3. UC-MSC Tendon Differentiation

UC-MSCs were plated at a density of 5 × 10^3^ cells/cm^2^. The differentiation medium was composed of DMEM (Invitrogen, Carlsbad, CA, USA), 10% fetal calf serum, 50 U/mL penicillin (Invitrogen, Carlsbad, CA, USA), 50 lg/mL streptomycin (Invitrogen, Carlsbad, CA, USA), 2 mM L-glutamine (Invitrogen, Carlsbad, CA, USA), and 5 ng/mL basic fibroblast growth factor (b-FGF-2) (PeproTech, Rocky Hill, New Jersey). UC-MSCs were cultured in tendon differentiation medium for 7, 14, and 21 days. The following experimental and control groups were analyzed:

- UC-MSCs cultured in the differentiation medium and exposed to PEMF for 2 hours (PEMF1 group), 4 hours (PEMF2 group), or 8 hours (PEMF3 group)
- UC-MSCs cultured in the differentiation medium without exposure to PEMF (CTRL1)
- UC-MSCs cultured in control medium (DMEM + 10% fetal calf serum, 50 U/mL penicillin/streptomycin, and 2 mM L-glutamine) and not exposed to PEMF (CTRL2)

Cultured cells were analyzed at day 0, day 7, day 14, and day 21.

PEMF stimulation was carried out as previously described by de Girolamo et al. [12]. UC-MSCs were exposed to PEMF generated by a pair of rectangular horizontal coils placed at opposite sites and composed of 1000 turns of copper wire. The culture plate was placed between the coils, keeping the plane of the coils parallel to the culture flasks. The coils were linked to a PEMF generator system (IGEA, Carpi, Italy) as previously described in several works [12, 13]. The system was able to produce a pulsed signal with a duration of 1.3 ms and a frequency of 75 Hz (yielding a 0.1 duty cycle). This corresponded to a peak intensity of the magnetic field of 1.5 mT.

### 2.4. Cell Apoptosis Analysis with Annexin V/Propidium Iodide

Apoptosis was analyzed at 7, 14, and 21 days of differentiation with annexin V FITC/propidium iodide (PI) staining (Thermo Fisher Scientific, Waltham, Massachusetts, USA). Apoptosis was expressed as a percentage of positive cells (annexin V+/PI− and annexin V+/PI+).

### 2.5. Immunofluorescence Analysis

Expression of the tenocyte markers scleraxis (Santa Cruz Biotechnology, Dallas, Texas, USA), collagen type I (Merck Millipore, Milano, Italy) and the proliferative marker PCNA (Santa Cruz Biotechnology, Dallas, Texas, USA) was assessed by immunofluorescence. Primary monoclonal antibodies were diluted at 1 : 200 in PBS-1% BSA and incubated with the sections for 2 h at room temperature. The secondary DyLight 488 antibody (KPL, Kirkegaard & Perry Laboratories, Maryland, USA), diluted at 1 : 100, was incubated for 1 h at room temperature. Stained sections were visualized with an Apotome fluorescence microscope (Zeiss). We collected digital images using a ×20 dry lens within 0–5 days after labeling.

### 2.6. Evaluation of Fluorescence Intensity

We have evaluated the difference of fluorescence intensity between groups using the ImageJ program. This software generated numerical semiquantitative evaluations corresponding to the mean of fluorescence intensity of each image examined. Ten cellular fields were randomly chosen among the different areas of migrated cells in each slide. Briefly, a point tool enables to mark different points on each image. With each “click,” the coordinates of the mark (*xx*, *yy*) and brightness values (0–255) are recorded in the data window. ImageJ brightness units are in a scale where 0 is pure black and 255 is pure white. Brightness values for each image were calculated as the arithmetical mean of all values in all fields recorded for that image. For each group, the mean fluorescence intensity of each marker was calculated and plotted in a graph. The difference in signal intensity allowed for evaluating the change in marker expression between the different culture conditions.

### 2.7. IL-10 and VEGF-A ELISA Analysis

IL-10 and VEGF-A expression in the culture medium was analyzed at 7, 14, and 21 days with a commercially available ELISA test, following the manufacturer's protocols (R&D Systems, Minneapolis, MN, USA).

### 2.8. Statistical Analysis

All data in the text and figures are provided as means ± standard deviation (SD). To compare the three different conditions, we have adopted the one-way ANOVA and Bonferroni adjustment. Statistical analysis was carried out with the statistical software package GraphPad Prism 5.0 (GraphPad Software).

## 3. Results (See Supplemental Results (Available Here))

### 3.1. UC-MSC Morphologic and Immunophenotypic Characterization

In primary cultures, typical spindle-shaped adherent cells were observed migrating from the UC tissue fragments and starting the colony formation approximately at day 14 after seeding. The UC immunophenotype was analyzed by flow cytometry. The majority of the collected UC cells showed positive expression of the main MSC markers CD73, CD90, and CD105, as well as of CD44 and CD29. Furthermore, they were negative for the typical hematopoietic marker CD34. The data also demonstrated the presence of HLA-ABC proteins and the absence of HLA-DR (data not shown).

### 3.2. Immunofluorescence Analysis

Immunofluorescence analysis revealed scleraxis, collagen type I (Col I), and proliferating cell nuclear antigen (PCNA) expression.

#### 3.2.1. Scleraxis

**Figure 1:**
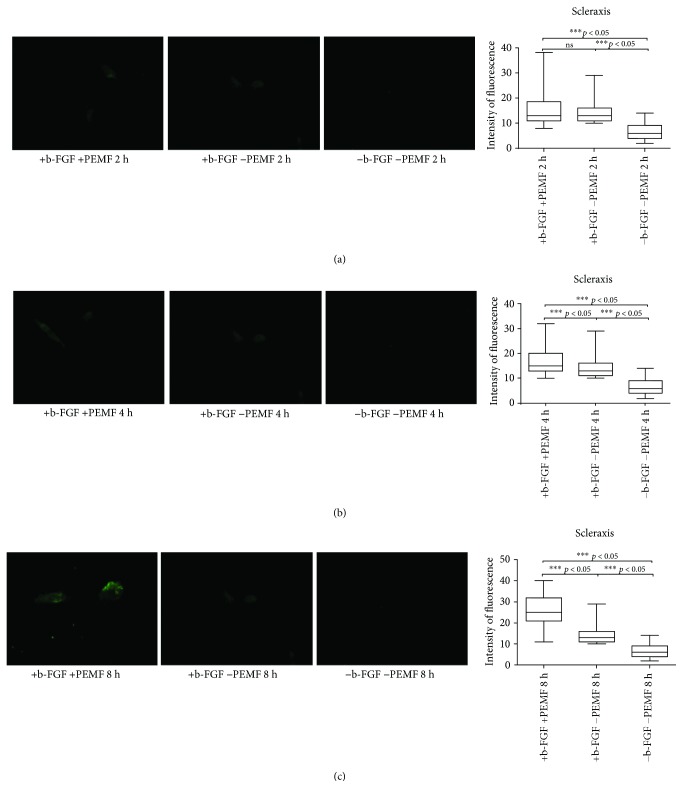
Scleraxis expression after 7 days of cell culture. (a) Immunofluorescent analysis for scleraxis expression and quantification of fluorescence intensity in UC-MSC cultures with and without PEMF exposure for 2 hours/day. (b) Immunofluorescent analysis for scleraxis expression and quantification of fluorescence intensity in UC-MSC cultures with and without PEMF exposure for 4 hours/day. (c) Immunofluorescent analysis for scleraxis expression and quantification of fluorescence intensity in UC-MSC cultures with and without PEMF exposure for 8 hours/day. ^∗∗∗^Indicates the *p*-vaule (probability value, statistical significance level <0.05) between two specific groups of study, ns: indicates a non significant value.

At day 7 (Figure 1), a greater presence of scleraxis-positive cells was observed in PEMF2 and PEMF3 groups. Moreover, there was a progressive increase in the absolute values of intensity of fluorescence with a prolonged exposure to PEMF (Figures 1(a)–1(c)).

**Figure 2:**
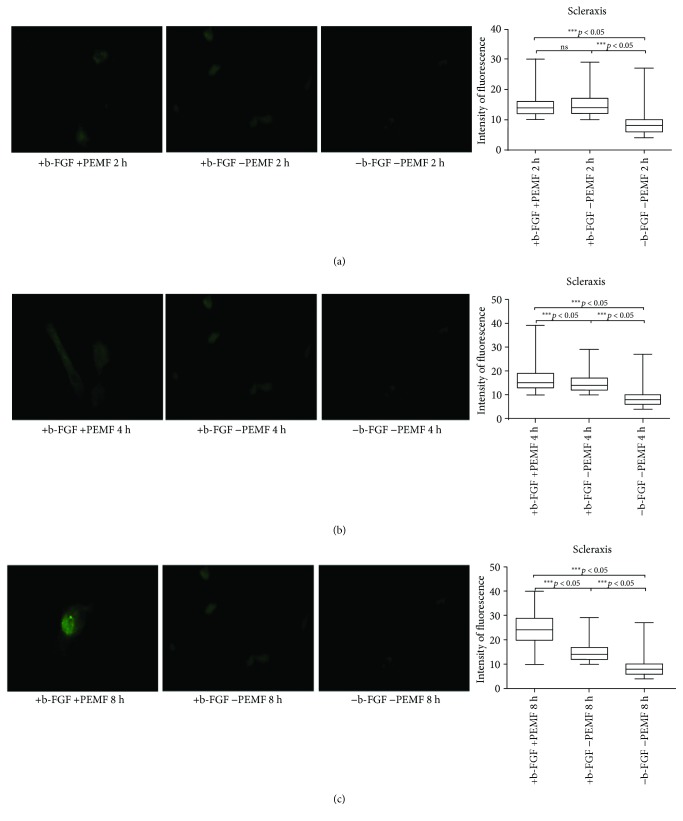
Scleraxis expression after 14 days of cell culture. (a) Immunofluorescent analysis for scleraxis expression and quantification of fluorescence intensity in UC-MSC cultures with and without PEMF exposure for 2 hours/day. (b) Immunofluorescent analysis for scleraxis expression and quantification of fluorescence intensity in UC-MSC cultures with and without PEMF exposure for 4 hours/day. (c) Immunofluorescent analysis for scleraxis expression and quantification of fluorescence intensity in UC-MSC cultures with and without PEMF exposure for 8 hours/day. ^∗∗∗^Indicates the *p*-value (probability value, statistical significance level <0.05) between two specific groups of study, ns: indicates a non significant value.

At day 14 (Figure 2), we obtained similar results (Figures 2(a)–2(c)).

**Figure 3:**
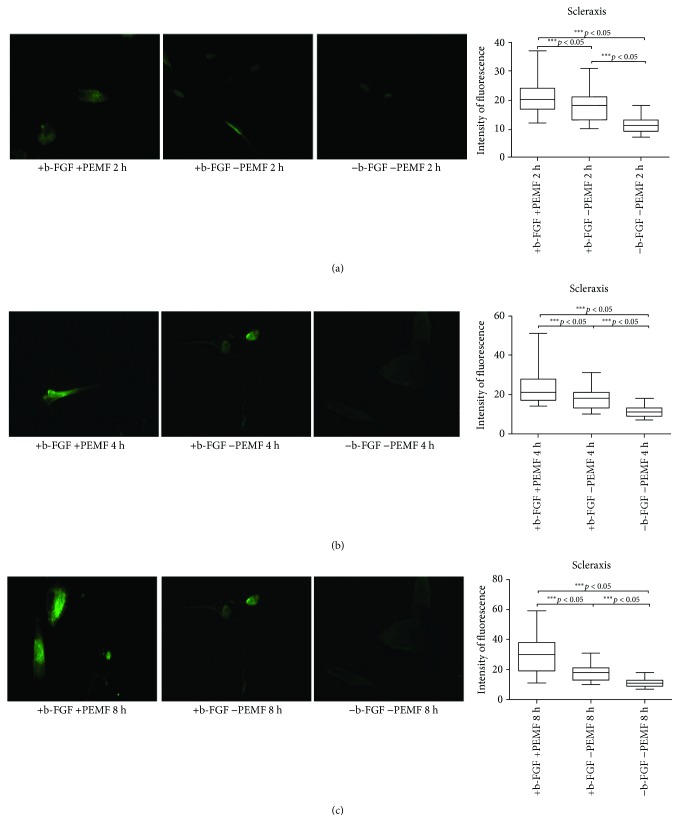
Scleraxis expression after 21 days of cell culture. (a) Immunofluorescent analysis for scleraxis expression and quantification of fluorescence intensity in UC-MSC cultures with and without PEMF exposure for 2 hours/day. (b) Immunofluorescent analysis for scleraxis expression and quantification of fluorescence intensity in UC-MSC cultures with and without PEMF exposure for 4 hours/day. (c) Immunofluorescent analysis for scleraxis expression and quantification of fluorescence intensity in UC-MSC cultures with and without PEMF exposure for 8 hours/day. ^∗∗∗^Indicates the *p*-value (probability value, statistical significance level <0.05) between two specific groups of study.

At day 21, there was a significant increase in the intensity of fluorescence in the experimental groups exposed to PEMF (Figure 3), especially in the PEMF3 group.

#### 3.2.2. Type I Collagen

**Figure 4:**
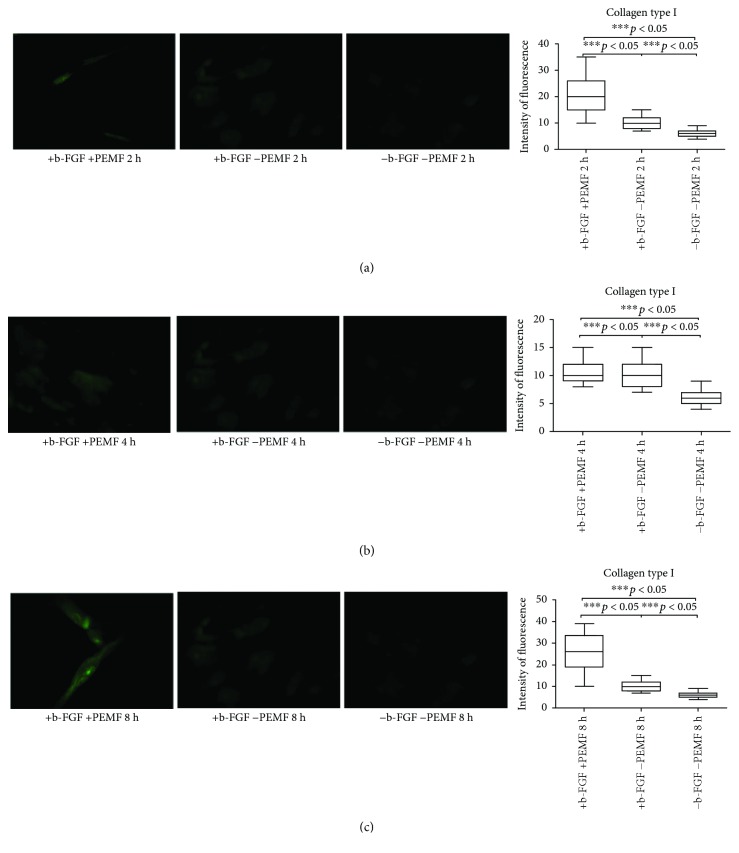
Type I collagen expression after 7 days of cell culture. (a) Immunofluorescent analysis for type I collagen expression and quantification of fluorescence intensity in UC-MSC cultures with and without PEMF exposure for 2 hours/day. (b) Immunofluorescent analysis for type I collagen expression and quantification of fluorescence intensity in UC-MSC cultures with and without PEMF exposure for 4 hours/day. (c) Immunofluorescent analysis for type I collagen expression and quantification of fluorescence intensity in UC-MSC cultures with and without PEMF exposure for 8 hours/day. ^∗∗∗^Indicates the *p*-value (probability value, statistical significance level <0.05) between two specific groups of study, ns: indicates a non significant value.

From day 7, samples exposed to PEMF showed significantly higher fluorescence intensity values than the two control conditions, with significant differences in PEMF2 and PEMF3 groups. The addition of the tenogenic growth factor FGF-2 to the culture medium determined greater expression of type I collagen (Figures 4(a)–4(c)).

**Figure 5:**
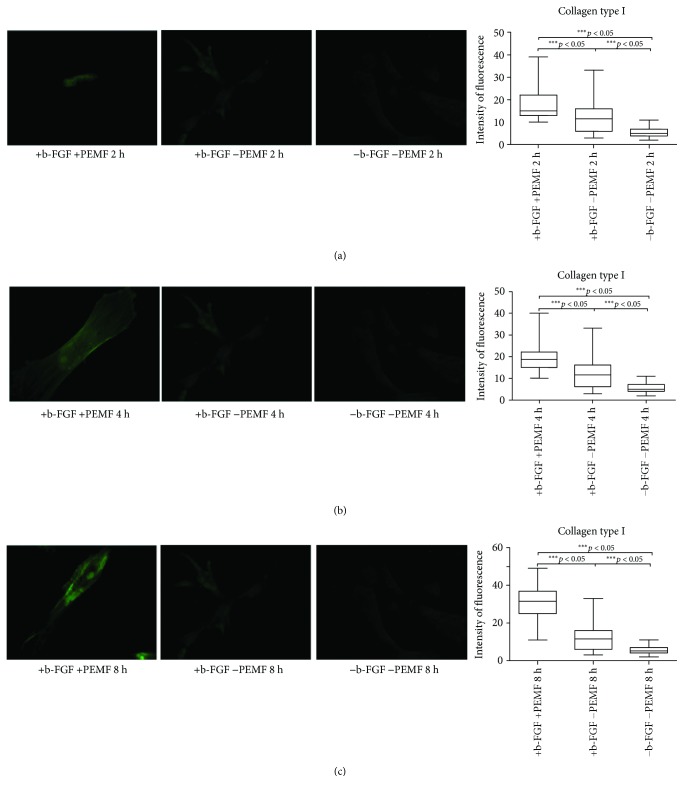
Type I collagen expression after 14 days of cell culture. (a) Immunofluorescent analysis for type I collagen expression and quantification of fluorescence intensity in UC-MSC cultures with and without PEMF exposure for 2 hours/day. (b) Immunofluorescent analysis for type I collagen expression and quantification of fluorescence intensity in UC-MSC cultures with and without PEMF exposure for 4 hours/day. (c) Immunofluorescent analysis for type I collagen expression and quantification of fluorescence intensity in UC-MSC cultures with and without PEMF exposure for 8 hours/day. ^∗∗∗^Indicates the *p*-value (probability value, statistical significance level <0.05) between two specific groups of study, ns: indicates a non significant value.

At day 14 (Figure 5), cells exposed to PEMF expressed significantly greater amounts of type I collagen. There was also a gradual increase in the absolute values of fluorescence intensity with time.

**Figure 6:**
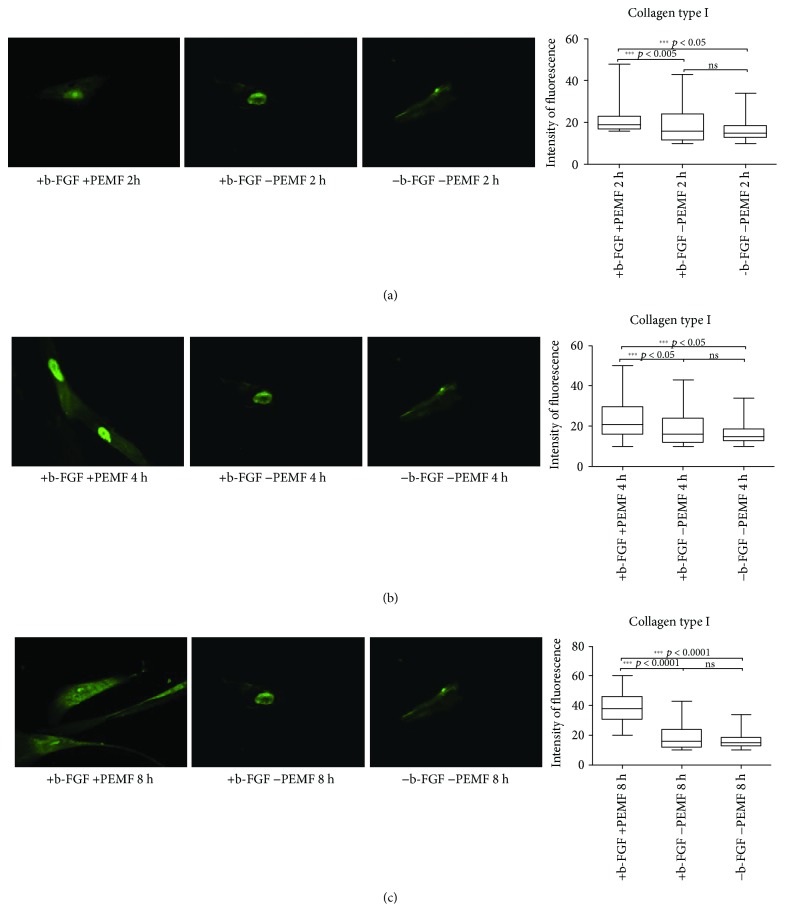
Type I collagen expression after 21 days of cell culture. (a) Immunofluorescent analysis for type I collagen expression and quantification of fluorescence intensity in UC-MSC cultures with and without PEMF exposure for 2 hours/day. (b) Immunofluorescent analysis for type I collagen expression and quantification of fluorescence intensity in UC-MSC cultures with and without PEMF exposure for 4 hours/day. (c) Immunofluorescent analysis for type I collagen expression and quantification of fluorescence intensity in UC-MSC cultures with and without PEMF exposure for 8 hours/day. ^∗∗∗^Indicates the *p*-value (probability value, statistical significance level <0.05) between two specific groups of study, ns: indicates a non significant value.

At the end of the three weeks (day 21) (Figure 6), fluorescence intensity was significantly greater in the 3 experimental groups exposed to PEMF. The 8-hour exposure protocol met the significance criteria with a *p* value < 0.0001 compared to the control groups. Moreover, FGF-2 did not affect type I collagen expression (Figures 6(a)–6(c)).

#### 3.2.3. PCNA

**Figure 7:**
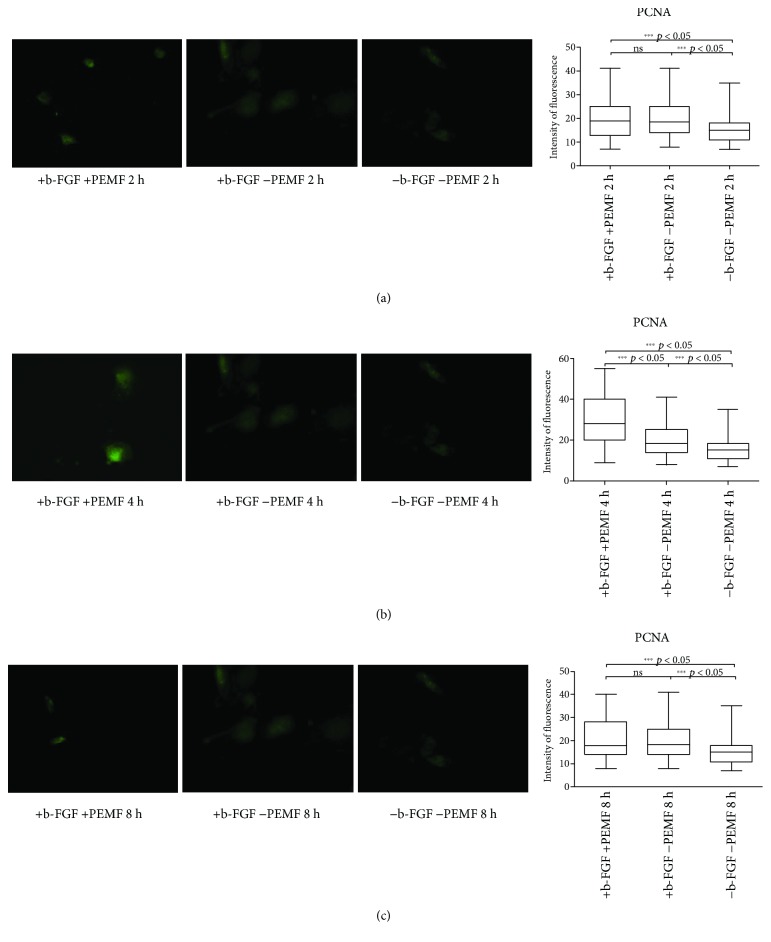
PCNA expression after 7 days of cell culture. (a) Immunofluorescent analysis for PCNA expression and quantification of fluorescence intensity in UC-MSC cultures with and without PEMF exposure for 2 hours/day. (b) Immunofluorescent analysis for PCNA expression and quantification of fluorescence intensity in UC-MSC cultures with and without PEMF exposure for 4 hours/day. (c) Immunofluorescent analysis for PCNA expression and quantification of fluorescence intensity in UC-MSC cultures with and without PEMF exposure for 8 hours/day. ^∗∗∗^Indicates the *p*-value (probability value, statistical significance level <0.05) between two specific groups of study.

After a week (day 7) of PEMF exposure, PCNA expression was not significantly higher than the PCNA expression in CTRL1, except for PCNA expression in the 4-hour protocol (Figure 7). Moreover, PEMF exposure or the presence of FGF-2 did not negatively affect PCNA expression (Figures 7(a)–7(c)).

**Figure 8:**
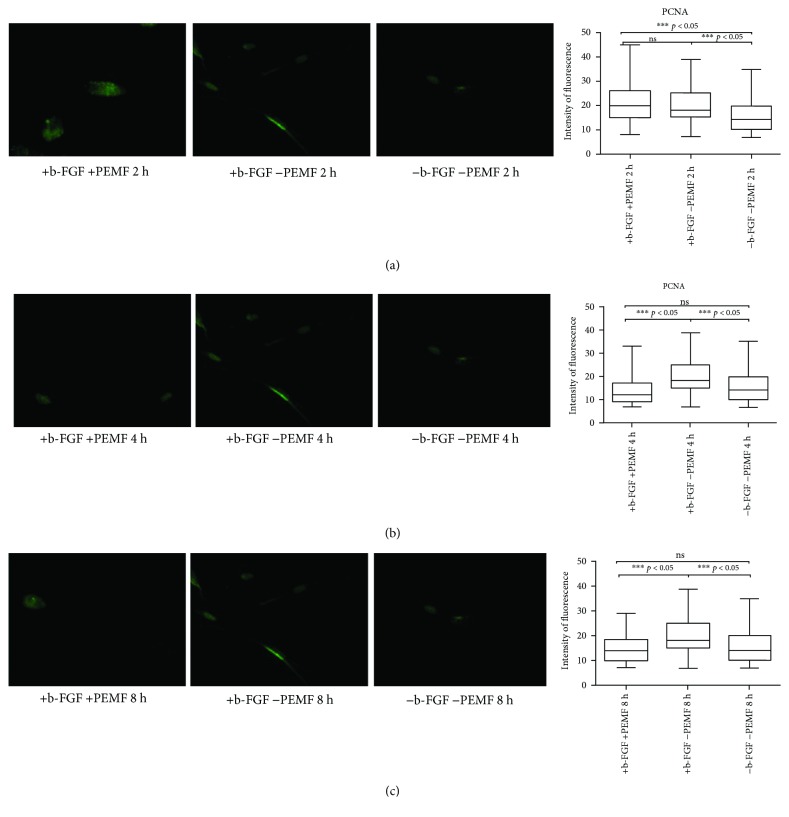
PCNA expression after 14 days of cell culture. (a) Immunofluorescent analysis for PCNA expression and quantification of fluorescence intensity in UC-MSC cultures with and without PEMF exposure for 2 hours/day. (b) Immunofluorescent analysis for PCNA expression and quantification of fluorescence intensity in UC-MSC cultures with and without PEMF exposure for 4 hours/day. (c) Immunofluorescent analysis for PCNA expression and quantification of fluorescence intensity in UC-MSC cultures with and without PEMF exposure for 8 hours/day. ^∗∗∗^Indicates the *p*-value (probability value, statistical significance level <0.05) between two specific groups of study.

At day 14, we observed a significantly greater fluorescence signal in CTRL1 than in the PEMF1 experimental group (Figure 8). There was no significant difference between PEMF3 and CTRL1 groups (Figures 8(a)–8(c)).

**Figure 9:**
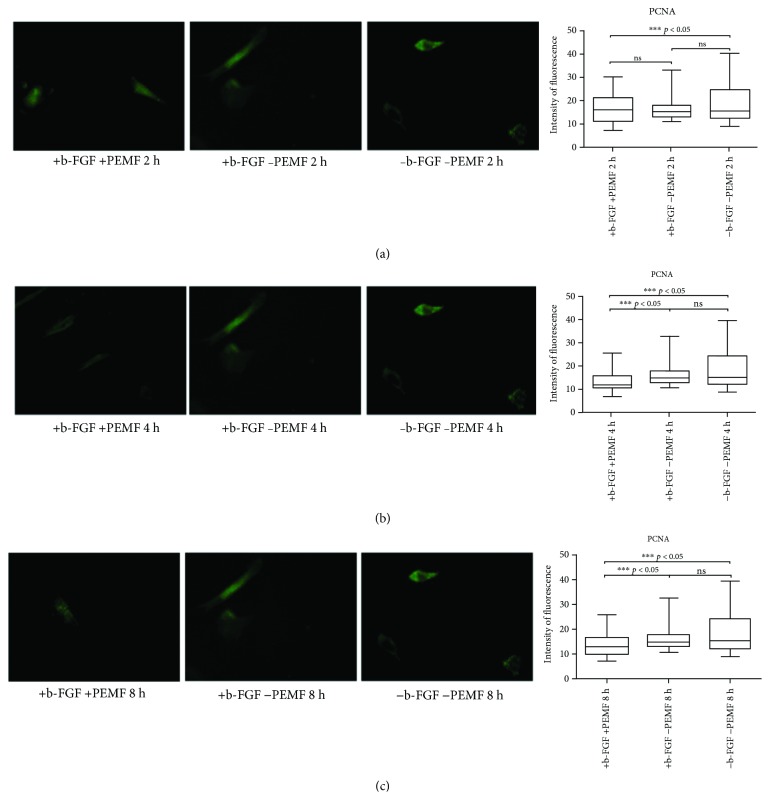
PCNA expression after 21 days of cell culture. (a) Immunofluorescent analysis for PCNA expression and quantification of fluorescence intensity in UC-MSC cultures with and without PEMF exposure for 2 hours/day. (b) Immunofluorescent analysis for PCNA expression and quantification of fluorescence intensity in UC-MSC cultures with and without PEMF exposure for 4 hours/day. (c) Immunofluorescent analysis for PCNA expression and quantification of fluorescence intensity in UC-MSC cultures with and without PEMF exposure for 8 hours/day. ^∗∗∗^Indicates the *p*-value (probability value, statistical significance level <0.05) between two specific groups of study, ns: indicates a non significant value.

At the end of the three weeks (day 21), PCNA expression was lower in PEMF groups (especially PEMF2 and PEMF3) than in the CTRL1 group (Figures 9(a)–9(c)).

### 3.3. IL-10 and VEGF Analysis

The immunoenzymatic test provided data on the presence of two major cytokines with modulating action on the immune and inflammatory response: interleukin-10 (IL-10) and vascular endothelial growth factor (VEGF).

**Figure 10:**
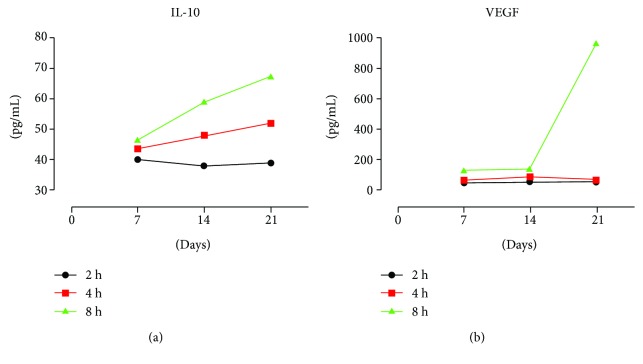
IL-10 and VEGF expression in UC-MSC cultures exposed to PEMF. (a) Analysis of IL-10 levels in cultures exposed to FGF-2 and PEMF at different PEMF expositions (2, 4, and 8 h/day) after 7, 14, and 21 days. (b) Analysis of VEGF levels in cultures exposed to FGF-2 and PEMF at different PEMF expositions (2, 4, and 8 h/day) after 7, 14, and 21 days.

Analysis of IL-10 production revealed a linear growth in PEMF2 and PEMF3 groups (Figure 10(a)). The curve slope increased with the progressive increase in PEMF exposure, with value shift between days 7, 14, and 21.  Figure 10(b) showed a remarkable increase in VEGF values starting from day 14 in the PEMF3 group.

**Figure 11:**
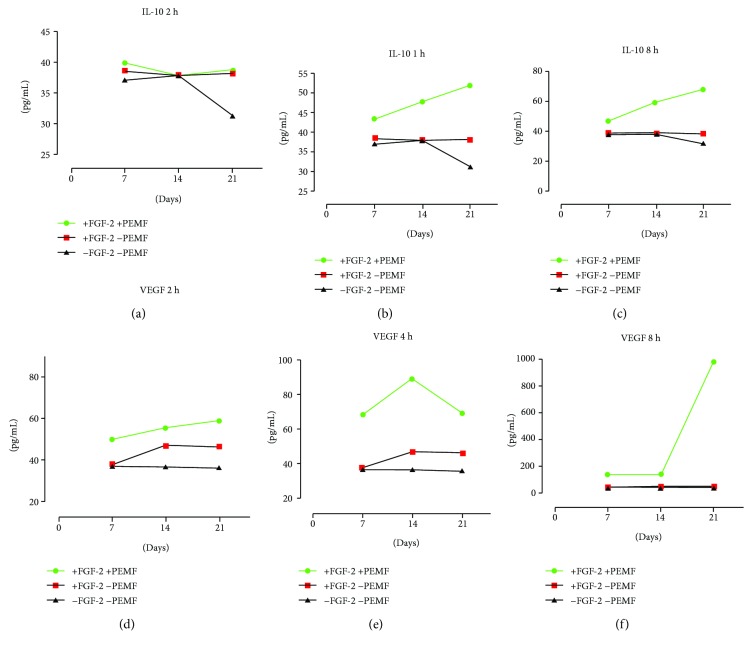
Comparison of IL-10 and VEGF expression between experimental and control groups. (a) Analysis of IL-10 levels in cultures exposed to FGF-2 and PEMF (2 h/day) compared to cultures exposed only to FGF-2 and cultures without PEMF and FGF-2 exposures after 7, 14, and 21 days. (b) Analysis of IL-10 levels in cultures exposed to FGF-2 and PEMF (4 h/day) compared to cultures exposed only to FGF-2 and cultures without PEMF and FGF-2 exposures after 7, 14, and 21 days. (c) Analysis of IL-10 levels in cultures exposed to FGF-2 and PEMF (8 h/day) compared to cultures exposed only to FGF-2 and cultures without PEMF and FGF-2 exposures after 7, 14, and 21 days. (d) Analysis of VEGF levels in cultures exposed to FGF-2 and PEMF (2 h/day) compared to cultures exposed only to FGF-2 and cultures without PEMF and FGF-2 exposures after 7, 14, and 21 days. (e) Analysis of VEGF levels in cultures exposed to FGF-2 and PEMF (4 h/day) compared to cultures exposed only to FGF-2 and cultures without PEMF and FGF-2 exposures after 7, 14, and 21 days. (f) Analysis of VEGF levels in cultures exposed to FGF-2 and PEMF (8 h/day) compared to cultures exposed only to FGF-2 and cultures without PEMF and FGF-2 exposures after 7, 14, and 21 days.

Comparing the different culture conditions (exposed to PEMF and not exposed to PEMF), we did not notice at 7 days any significant differences in IL-10 expression between PEMF-exposed groups and control groups (Figure 11). In the CTRL2 group (without PEMF and FGF), there were stable values of IL-10 from day 7 to day 14, followed by a fall at day 21. In the CTRL1 group (with FGF but without PEMF), stationary concentrations were found at all culture time points (i.e., at 7, 14, and 21 days). On the other hand, in the PEMF-exposed cultures (i.e., UC-MSC cultures with PEMF and FGF), a significant increased release of IL-10 was observed with the conditions of 4 and 8 hours per day of PEMF exposure (PEMF2 and PEMF3 groups) at day 14 and, more markedly, at day 21.

VEGF expression was higher in all PEMF-exposed cultures (PEMF1, PEMF2, and PEMF3) than in control groups. In the control group exposed to FGF-2 (CTRL1 group), the presence of FGF positively influenced VEGF expression with time, with values inferior to those observed in the PEMF-exposed groups. The largest deviation in VEGF production between PEMF-exposed cultures and control cultures not exposed to PEMF (CTRL1 and CTRL2 groups) was observed to be related to the group exposed to PEMF for 8 hours daily (PEMF3) (Figure 11(f)).

### 3.4. Cell Viability Analysis

**Figure 12:**
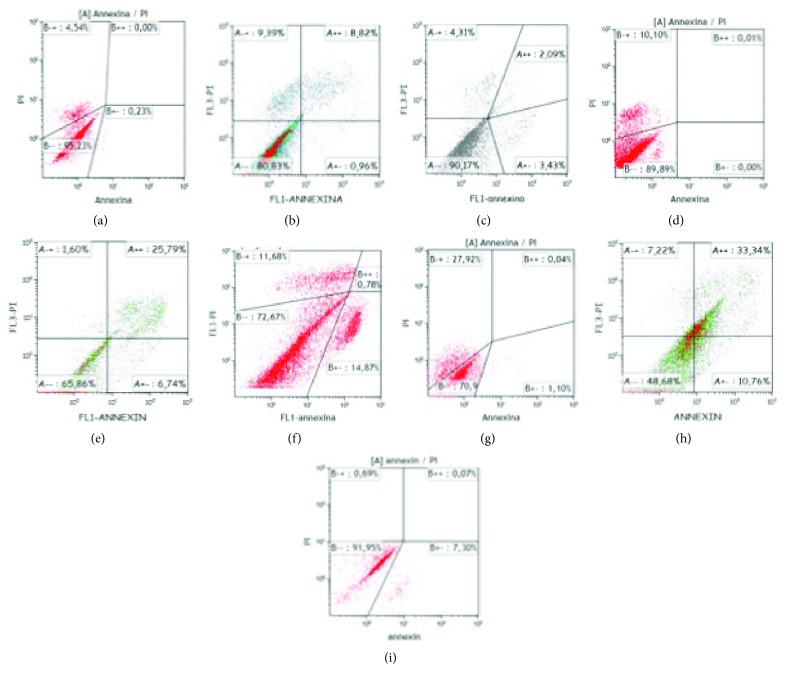
Evaluation of cell mortality in UC-MSC cultures exposed to PEMF. (a) FACS analysis of annexin V/propidium iodide in cultures exposed to FGF-2 and PEMF (2 h/day) after 7 days. (b) FACS analysis of annexin V/propidium iodide in cultures exposed to FGF-2 and PEMF (4 h/day) after 7 days. (c) FACS analysis of annexin V/propidium iodide in cultures exposed to FGF-2 and PEMF (8 h/day) after 7 days. (d) FACS analysis of annexin V/propidium iodide in cultures exposed to FGF-2 and PEMF (2 h/day) after 14 days. (e) FACS analysis of annexin V/propidium iodide in cultures exposed to FGF-2 and PEMF (4 h/day) after 14 days. (f) FACS analysis of annexin V/propidium iodide in cultures exposed to FGF-2 and PEMF (8 h/day) after 14 days. (g) FACS analysis of annexin V/propidium iodide in cultures exposed to FGF-2 and PEMF (2 h/day) after 21 days. (h) FACS analysis of annexin V/propidium iodide in cultures exposed to FGF-2 and PEMF (4 h/day) after 21 days. (i) FACS analysis of annexin V/propidium iodide in cultures exposed to FGF-2 and PEMF (8 h/day) after 21 days.

Cell mortality data at day 7 showed 95.23% survival rate for PEMF1, 80.83% for PEMF2, and 90.17% for PEMF3, with the highest percentage of necrosis and apoptosis (PI-positive cells or PI- and annexin-positive cells) found in the PEMF2 group (Figure 12).

At day 14, there was a decrease in the percentage of surviving cells in all the three experimental groups, particularly in PEMF3, in which, besides a decrease of about 17.5% of the live cells, there was a percentage of 14.87% cells in early apoptosis (Figures 12(d)–12(f)). The lowest percentage of cell survival was reported in 4 h exposure protocol (65.86%) with 25.79% positive double cells.

At the final time point at three weeks (day 21), we found a further decrease in cell survival in the 2- and 4-hour protocols (19% and 17%, resp.), consensually with the negative trend already recorded in the previous analyses (Figures 12(g)–12(i)). In the 8-hour culture, however, there was a very high percentage of surviving cells (91.95%) compared to that at day 14 (72.67%).

## 4. Discussion

The main finding of this study is that the exposure to PEMF (of 1.5 mT and 75 Hz) provides a biophysical stimulus, which significantly enhances the in vitro tendon commitment of human UC-MSCs. Thus, exposure to PEMF may represent an alternative biologic scenario of MSC preconditioning toward the tenogenic pathway.

Moreover, our study suggests that the combination of PEMF and allogeneic human UC-MSCs may represent a possible therapeutic alternative for tendon tissue engineering. These results, indeed, may have a clinical relevance for future preclinical and clinical models of tendon regeneration and rotator cuff reconstructive surgery.

The role of MSCs in tendon repair and regeneration is still a debated topic in literature. Several observations from different authors have questioned the possible positive influence of the direct application of undifferentiated MSCs at a tendon lesion site. Gulotta et al. [5] have shown that the use of bone marrow-derived MSCs in the repair of rotator cuff lesions in rats did not improve the healing potential, suggesting that the simple enrichment with MSCs at the lesion site was not sufficient for tissue regeneration. A similar observation was confirmed by Okamoto et al. [17] who showed better results with the bone marrow concentrate when compared to a purified suspension of precultured undifferentiated MSCs in a healing model of Achilles tendon lesion in rats. More recently, Huang et al. [18] observed improvements in the healing of rat Achilles tendon rupture when MSCs were preactivated in hypoxic conditions. This result suggests the need for a supplemental signal for MSCs to be efficiently “preconditioned” toward the tenogenic pathway and to positively interfere with the tendon and enthesis repair, as also recently indicated by Chamberlain et al. [19]. Thus, this concept may represent a possible answer to the putative “elusive” role of MSCs in tendon [5]. Indeed, the hypoxic culture represents an alternative method to activate MSCs, leading them toward a specific chondrogenic pathway. Moreover, the whole bone marrow may include some of the “stem cell niche” that may produce biologic factors able to activate in situ resident MSCs as well as the few MSCs present in the bone marrow aspirate. Moving further, few studies have demonstrated that also the gene transfer procedure may be considered a solution for MSC preconditioning, through the transfection of scleraxis [7] or membrane type 1 matrix metalloproteinase [20]. Very recently, the association of the cortical allogeneic demineralized bone matrix and MSCs has also given promising results in a rat model of supraspinatus lesion. This is in line with the concept of “MSC preconditioning,” due to the ability of demineralized bone matrix to release growth factors (i.e., bone morphogenetic proteins) [21]. This study, nevertheless, introduces a concept of “fine tuning” effect of MSCs on the microenvironment, in line with a different in vitro study by Rehmann et al. who demonstrated the increased production of tenogenic proteins by MSCs in the presence of a specific bioactive scaffold [22].

In summary, one of the key factors to promote MSC tenogenic potential resides in the “preactivation” or “preconditioning” of these progenitor cells, by direct intracellular stimulation (i.e., gene transfection) or by exerting a positive stimulus on the MSC microenvironment.

From this perspective, PEMF may represent a promising and cost-efficient alternative. Indeed, the positive role of PEMF in the mesenchymal healing process is well known in literature [10]. Several studies have shown that PEMF may activate several cellular mediators, such as adenosine receptors [23], which are linked to the inhibition of the activation of transcription factor NF-*κ*B. Furthermore, PEMF seems to interact with the ligand-independent activation of the epidermal growth factor receptor (EGFR) and other members of the receptor tyrosine kinase family that are involved in the activation of the MAPK (mitogen-activated protein kinase)/ERK (extracellular signal-regulated kinase) pathway and in the stimulation of the intracellular mitogenic pathways [10, 24]. Wnt signaling proteins (Wnt1/lipoprotein 247 receptor-related protein 5 (LRP5)/beta-catenin) may also be involved in the PEMF mechanism, outlining the possible anabolic role of PEMF toward mesenchymal cell proliferation. Further studies are needed to better clarify the still “hidden” mechanisms activated by PEMF stimulation. Nonetheless, these observations suggest a possible intracellular anabolic role of PEMF exposure. This effect has also been “indirectly” observed even for the tendon repair process. In a preclinical animal model, Huegel et al. [8] showed a positive result of PEMF exposure (for 1, 3, or 6 hours daily) in a rat model of acute supraspinatus injury and repair, while Tucker et al. [11] observed an improved early tendon healing following PEMF exposure in a similar preclinical model of rotator cuff repair. The in vitro effects of PEMF on tendon cells have also been extensively examined, and several insights have been clarified. Indeed, PEMF may have a positive effect in promoting tenocyte gene expression and myoblast differentiation as observed by Liu et al. [9]. These authors demonstrated increased expression of growth factor genes in a human rotator cuff tenocytic cell line and an increased myotube formation in a murine myoblast cell culture. Previously, de Girolamo et al. [12, 13] had already confirmed a positive influence of PEMF scleraxis, type I collagen, IL-6, IL-10, and VEGF expression in the human tenocytic cell line derived. Thus, a positive role of PEMF toward tendon commitment may be considered, summarizing the effect as (i) reduction of the catabolic effects of proinflammatory cytokines (such as IL-1) and (ii) promotion of ECM production, anabolic cytokine release, and cell proliferation through the increased expression of scleraxis, VEGF, and COL1A1 genes and the release of IL-6, IL-10, and TGF-*β*.

The present study is aimed at verifying the possible positive role of PEMF on a specific MSC source, namely, the allogeneic human UC-MSCs. Indeed, the umbilical cord effectively represents a fascinating cell source and a hypothetical therapeutic alternative for tendon and enthesis repair. MSCs derived from the UC maintain a phenotype that recapitulates some features with embryonic stem cells, having a fetal origin, but without some crucial ethical concerns. Indeed, UC is considered a discarded material, and it does not involve any donor site morbidity. Thus, it represents a good candidate for any “one-stage” procedures. Furthermore, it may be stored in any certified stem cell factory in great amount, being available at demand, and thus it may become an “off-the-shelf” cell population ready to be used for different reconstructive orthopaedic procedures [15, 16]. For all these reasons, UC-MSCs may represent an appealing alternative as a cell source for tendon healing in a hypothetical preclinical and clinical setting. A further “collateral suggestion” of the positive role of umbilical cord-derived cells came recently from the preclinical xenogenic study of Park et al. who showed the partial healing of a full-thickness subscapularis tendon tear in rabbits by a simple injection of human umbilical cord blood- (UCB-) derived mesenchymal stem cells.

In our in vitro model, we observed a positive influence of PEMF on the tenogenic differentiation of UC-MSC cultures. Indeed, scleraxis and type I collagen expression was significantly increased following PEMF exposure. We also identified the optimal time interval to increase tendon marker expression and anabolic cytokine release. Indeed, we applied different time exposures to the UC-MSC cultures. Albeit all types of exposures led to positive results, a greater difference was observed in the PEMF3 group, compared to control groups. Thus, these results suggest the 8-hour exposure as the best PEMF exposure to stimulate UC-MSC tenogenic potential. This result may also have a clinical relevance in the perspective of the use of PEMF for improving the immediate postoperative healing of tendon repairs.

We also observed reduced PCNA expression at the longest experimental time point. This result is consistent with a predictable reduction in cell proliferation at day 21, due to the presence of multiple differentiation stimuli in the culture setting, where the combined actions of PEMF, FGF-2, and the soluble cytokines released by the UC-MSCs take place. This observation seems to be in apparent contrast with the previously described anabolic effect of PEMF on mesenchymal cell lines. However, these data prove that a tenogenic commitment at the end of the PEMF exposure physiologically leads to a decrease in cell proliferation. Moreover, reduced PCNA expression was not observed at the early time points (i.e., at days 7 and 14) of the culture exposed to PEMF (8 h/day), suggesting that the exposure to PEMF might not impair the very early healing phase of the tendon repair process, when a proliferative state of the cell is desirable.

In our study, an increased release of IL-10 and VEGF was detected at any time point during PEMF exposure, albeit the increase was more significant at day 21 and after 8 hours of exposure. This observation emphasizes the possible tenogenic role of PEMF exposure. Indeed, the effect of the PEMF seems to induce both a direct cellular commitment, as shown by the increased expression of scleraxis and type I collagen, and a concomitant indirect tenogenic activation by promoting the release of soluble immunoregulatory cytokines such as IL-10 and growth factors such as VEGF involved in tendon healing. The pattern of IL-10 and VEGF release parallels the tenogenic marker expression, further confirming the exposure of 8 h/day as the better “anabolic protocol” for the UC-MSCs.

Thus, the clinical relevance of our results may be hypothesized. Firstly, our research confirms the positive effect of PEMF on MSC tenogenic commitment. This perception may suggest a broader application of this technique after common reconstructive orthopaedic procedures as well as rotator cuff reconstruction. Indeed, during the early postoperative period, it is presumable that PEMF may exert both an anti-inflammatory and anabolic effect at the surgical site, by interacting with the endogenous tissue-specific resident MSCs, as suggested by Viganò et al. [25]. Evidence has also been recently shown by Huegel et al. [8] in a preclinical study of acute rotator cuff injury. These authors showed positive effects of PEMF on the repair site after transosseous suture of acute supraspinatus transection in rats, observing an increase in physical and histological properties of the newly formed tissue. Similar results have been obtained by Tucker et al. in a preclinical rat rotator cuff repair model, where an improved tendon healing was noticed at the early phase of the repair [11]. Further large-scale clinical studies may allow for confirming these results with hopefully promising clinical results.

Secondly, a possible therapeutic strategy may be envisioned combining the direct addition of allogeneic UC-MSCs at the tendon repair site (i.e., the rotator cuff insertion) followed by an immediate postoperative PEMF treatment. This solution may represent a possible alternative to improve tendon repair by cell therapy, overcoming the unsuccessful paradigm of uncommitted undifferentiated MSC addition. Indeed, the presence of biophysical stimuli driven by PEMF may lead the UC-MSCs to a tenogenic commitment, realizing the concept of “MSC preconditioning” by a fine tuning of the cell microenvironment. Furthermore, UC-MSC tenogenic activation by PEMF is much more economically sustainable than the gene transfection described by Gulotta et al. [7, 20].

Lastly, a different therapeutic paradigm may be further hypothesized, conceiving MSCs as a “biological factory” to be used strictly in an in vitro condition. In this setting, a “tenogenic” secretome may be obtained following UC-MSC exposure to PEMF. This hypothetical “tenogenic secretome” could be extracted and subsequently administered locally at the surgical repair site at different time points, exerting a positive effect on tendon repair. This theory may actually represent an innovative perspective that would eliminate the concerns on MSC delivery, and it would introduce the new horizon of the “paracrine therapy” by means of PEMF-exposed UC-MSCs. In the presence of local stem cell factories, a broader application of this concept may be envisioned as an off-the-shelf cell-free one-step therapy for tendon regeneration. The potential of this hypothesis has recently been suggested by Sevivas et al. in preclinical models of rat rotator cuff tears [26, 27]. These authors observed a decreased fatty degeneration and an increased muscular mass improved after local injection of human MSC secretome [26]. The authors also noticed an anabolic effect of MSC secretome on human tendon cells in vitro and an improvement in enthesis reconstruction after implantation of the scaffolds seeded with human tendon cells pretreated with human MSC secretome [27]. An imperative attention to this new approach involving MSC microvesicles is needed as it may represent one of the ultimate evolutions of the use of stem cells for tissue engineering [28].

However, our study has several limitations. First of all, it suggests a therapeutic protocol based on in vitro observations. Indeed, the *in vivo* microenvironment at the lesion site and the presence of the natural inflammatory reaction may cause possible variations with regard to the positive effects of PEMF described in the present work. Albeit several observations in literature are in line with our results [8, 11, 25], the promising effect and the efficacy of the association of PEMF and UC-MSCs need to be further clarified in preclinical studies before any hypothetical clinical applications. Furthermore, UC-MSC culture was performed in a monolayer setting while, during any *in vivo* conceivable use of the “stem cell therapy,” a scaffold is needed to host the cells and to maintain the cells at the lesion site. Thus, a confirmation of the PEMF effect on UC-MSCs in a tridimensional setting may be desired before a direct preclinical application.

## 5. Conclusion

In this in vitro study, we observed that the PEMF exposure generates a biophysical preconditioning effect on UC-MSCs, promoting the expression of tenogenic markers and anabolic cytokines involved in tendon regeneration. Thus, PEMF effectively represents a potential key factor to promote MSC tenogenic commitment.

This effect is greater when a prolonged exposure of 8 hours per day is carried out. The positive results are more pronounced at 21 days, suggesting a PEMF time-dependent action on the UC-MSC tenogenic pathway.

Confirming the anabolic properties of PEMF on MSC tenogenic commitment, our study may have a clinical relevance. Indeed, the newly described tenogenic effects, along with the well-known PEMF chondrogenic properties, suggest a possible large-scale clinical application of this technique as an adjuvant device in the early postoperative period of tendon surgical repair. Furthermore, the combined use of PEMF and allogeneic UC-MSCs may be hypothesized as a future therapeutic perspective for tendon tissue engineering, both by a direct in situ cell delivery or by injection of the secretome derived from PEMF-preconditioned UC-MSCs, to improve tendon regeneration and enthesis restoration.

## Supplementary Materials

Supplementary Materials Supplementary Results: this section clarifies the details of the numerical values of the different parameters depicted in the figures and previously described in Results of the main manuscript.
