# Corrigendum to “Pulsed Electromagnetic Fields Improve Tenogenic Commitment of Umbilical Cord-Derived Mesenchymal Stem Cells: A Potential Strategy for Tendon Repair—An In Vitro Study”

**DOI:** 10.1155/2019/9761573

**Published:** 2019-05-02

**Authors:** Antonio Marmotti, Giuseppe Maria Peretti, Silvia Mattia, Laura Mangiavini, Laura de Girolamo, Marco Viganò, Stefania Setti, Davide Edoardo Bonasia, Davide Blonna, Enrico Bellato, Giovanni Ferrero, Filippo Castoldi

**Affiliations:** ^1^Department of Orthopaedics and Traumatology, University of Turin, Torino, Italy; ^2^Molecular Biotechnology Center, University of Turin, Torino, Italy; ^3^IRCCS Istituto Ortopedico Galeazzi, Milano, Italy; ^4^Department of Biomedical Sciences for Health, University of Milan, Milano, Italy; ^5^IGEA SpA Clinical Biophysics, Carpi, Modena, Italy

In the article titled “Pulsed Electromagnetic Fields Improve Tenogenic Commitment of Umbilical Cord-Derived Mesenchymal Stem Cells: A Potential Strategy for Tendon Repair—An In Vitro Study” [1], there was an error in the affiliation details of Drs. Laura Mangiavini, Laura de Girolamo, and Marco Viganò, as they were wrongly affiliated to “^4^Department of Biomedical Sciences for Health, University of Milan, Milano, Italy.” However, they should be affiliated to “^3^IRCCS Istituto Ortopedico Galeazzi, Milano, Italy.” The corrected authors' list and affiliations are shown above.
