# Supplementary material for: Pulsed Electromagnetic Fields Improve Tenogenic Commitment of Umbilical Cord-Derived Mesenchymal Stem Cells: A Potential Strategy for Tendon Repair—An In Vitro Study
Source: Stem Cells Int. 2018 Jul 30;2018:9048237. doi: 10.1155/2018/9048237 (PMC6091420; doi:10.1155/2018/9048237)

Methods and Materials

Approvals were obtained both from the Ethical Committee of MBC (Molecular Biotechnology Center), University of Turin, and from the Ethical Committee of Mauriziano Hospital, Turin (Italy); protocol number: CS792 approved on January 11th 2016

UC collection and processing

Fresh UC samples from 15 women with healthy pregnancies were recovered during caesarean deliveries from the Department of Obstetrics and Gynecology of Mauriziano Hospital (Turin, Italy). The 7 UC samples were collected in a phosphate-buffered saline (PBS) (Invitrogen, Carlsbad, CA, USA) transfer medium containing 200 mg/100mL ciprofloxacin (Bayer, Milan, Italy), 500 IU heparin (Pharmatex, Milan, Italy). After transferring under a sterile laminar flow cell culture hood, the cord length and weight were estimated and the UC was washed in PBS in order to eliminate the traces of contaminant red blood cells. The UC was cut into 3 cm long segments, which were subsequently cut longitudinally and split open to expose the inner surface. The UC segments were then manually minced into very small cuboidal fragments (4–7 mm length). The umbilical cord fragments were seeded in 60 cm^2 Petri dishes together with the same expansion medium in which they have been minced. This mesenchymal stem cells expansion medium contained Dulbecco's Modified Eagle Medium/F-12 (D-MEM) (Invitrogen, Carlsbad, CA, USA) enriched with 5% human platelet lysate obtained from healthy donors, 10% Fetal Bovine Serum (FBS), 1X penicillin/streptomycin (Invitrogen, Carlsbad, CA, USA), 1X sodium pyruvate (Invitrogen, Carlsbad, CA, USA), 1X nonessential amino acids (Invitrogen, Carlsbad, CA, USA), 500 IU heparin (Pharmatex, Milan, Italy).
The small UC fragments were distributed into different 60 cm^2 Petri dishes (approximately 40–45 fragments/Petri dish) and incubated in the MSC expansion medium at 37°C in a humidified atmosphere with 5% CO_2 (day 0). Fragments of UC were left undisturbed in culture and monitored for up to 2 weeks to allow for migration and adherence of MSC in the plastic dishes and subsequent identification of the adherent MSC population .

Culture and immunophenotypic characterization of UC-MSC

After 2 weeks from the initial seeding (day 14), the UC tissue was removed and adherent cells were allowed to expand for 2 additional weeks.
Forty percent of the medium was changed every 3-4 days. After this time period (day 28), the adherent cells (P0) were trypsinized, centrifuged at 1200 rpm for 10 min, resuspended in MSC expansion medium, and replated for one consecutive expansion step at a density of 100–200 cells/cm^2, until full confluence was reached (P1). Cell confluence at P1 was reached after approximately 14 days (day 42).
At the end of P1 passage (day 42), the living cells were counted by trypan blue dye exclusion.
Immunophenotype of the expanded UC-MSCs was done by flow cytometry analysis at P1. 1,5×10E6 UC-MSC were used for flow cytometry. The following antibodies were used: CD90-Peridinin Chlorophyll Protein (PerCP)-cyanine dye Cy5.5 (Biolegend, San Diego, CA), CD105- fluorescein isothiocyanate (FITC) (Biolegend, San Diego, CA), CD73-Allophycocyanin (APC) (BD Biosciences, San Jose, CA), CD34-phycoerythrin (PE) (BD Biosciences, San Jose, CA), HLA-DR-FITC (BD Biosciences, San Jose, CA), HLA-PerCP (BD Biosciences, San Jose, CA), HLA-ABC-PE, CD29- APC (BD Biosciences, San Jose, CA), CD44-Alexa Fluor (Cell Signaling Technology, Danvers, MA), PE-conjugated antimouse immunoglobulin G (IgG) (Southern Biotechnology Associates, Birmingham, Alabama, USA), isotypematched IgG-FITC (Biolegend, San Diego, CA), IgG-PE (Biolegend, San Diego, CA) and IgG-PE-Cy5 (Biolegend, San Diego, CA) control

antibodies. Analysis was performed on a FACScan (Becton Dickinson (BD), Buccinasco, Italy) for at least 10.000 events and using CellQuest software (BD, Buccinasco, Italy).

Tendon differentiation of UC-MSC

For the tendon differentiation, the UC-MSC were plated at a density of 5x10E3 cells/cm^2. The differentiation medium was composed of DMEM (Invitrogen, Carlsbad, CA, USA), 10% fetal calf serum, 50 U/ml Penicillin (Invitrogen, Carlsbad, CA, USA), 50 lg/ml streptomycin (Invitrogen, Carlsbad, CA, USA), 2 mM L-glutamine (Invitrogen, Carlsbad, CA, USA) and 5 ng/ml basic fibroblast growth factor (b-FGF) (PeproTech, Rocky Hill, New Jersey). The umbilical cord mesenchymal cells were cultured in tendon differentiation medium for 7, 14, 21 days (in chamber slides, in order to optimize the final immunofluorescence analysis).

The following cell cultures were set up:

- UC-MSC cultured in presence of the differentiation medium and exposed to PEMF for 2 hours (PEMF1), 4 hours (PEMF2) and 8 hours (PEMF3).
- UC-MSC cultured in presence of the differentiation medium without exposure to PEMF
- UC-MSC cultured in presence of control medium (DMEM + 10%fetal calf serum, 50 U/ml Penicillin /Streptomycin, 2 mM L-glutamine).

The cultured cells were analyzed at day 0, day 7, day 14 and day 21.

In this way, it was evaluated the effect of electromagnetic fields at different time point to allow for identifying the better setup for the tenogenic differentiation. PEMF stimulation was carried out with the same procedure as described by De Girolamo et al [4]. The UC-MSC were exposed to PEMF generated by a pair of rectangular horizontal coils placed opposite each other and made of 1,000 turns of copper wire. The culture plate was placed between the coils, keeping the plane of the coils parallel to the culture flasks, in a direction direct inside the incubator. The coils were linked to a generator system of PEMF (IGEA, Carpi, Italy) previously described in several in-vitro works [4] [5]. The system was able to produce a pulsed signal with duration of 1.3 ms and frequency of 75 Hz (yielding a 0.1 duty cycle). This corresponded to a peak intensity of the magnetic field of 1.5 mT.

Cell apoptosis analysis with annexin V/propidium iodide

Apoptosis induced by PEMF treatment was analyzed 7, 14, 21 days of differentiation, through the use of flow cytometry, using Annexin V FITC /Propidium iodide (PI) staining (Thermo Fisher Scientific, Waltham, Massachusetts, USA). Apoptosis was expressed as a percentage of positive cells annexin V +/PI- and annexin V +/PI +.

Immunofluorescence analysis

Expression of tenocyte markers scleraxis (Santa Cruz Biotechnology, Dallas, Texas, USA) and collagen type I (Merck Millipore, Milano, Italy), and proliferative marker PCNA (Santa Cruz Biotechnology, Dallas, Texas, USA) was assessed using immunofluorescence techniques. The primary monoclonal antibodies were diluted 1:200 in PBS-BSA1% and incubated with the sections for 2h at room temperature. The secondary dylight 488 antibody (KPL, Kirkegaard & Perry Laboratories, Maryland, USA), diluted 1:100, was incubated for 1h at room temperature. The stained sections were visualized with an Apotome fluorescence microscope (Zeiss). We collected digital images using a ×20 dry lens within 0–5 days after labeling.

Evaluation of fluorescence intensity

We have evaluated the difference of intensity of fluorescence between different constructs, using ImageJ program. This software generated numerical semi-quantitative evaluations corresponding to the mean fluorescence intensity of each image examined. Ten cellular

fields were randomly chosen among the different areas of migrated chondrocyte in each slide. Briefly, a point tool enables the marking of locations on an image. With each "click" the coordinates of the mark (xx, yy) and brightness values (0-255) are recorded in a data window. ImageJ brightness units are in a scale where 0 = pure black and 255 = pure white. Brightness values for each image were calculated as the arithmetical mean of all values in all fields recorded for that image. For each construct, mean fluorescence intensity of each marker was calculated and plotted as a graph. The difference in intensity allowed for evaluating the change in markers expression between the different culture conditions.

Elisa test of IL-10 and VEGF-A

IL-10 and VEGF-A soluble levels present in the culture medium were analyzed 7, 14, 21 days of differentiation for each PEMF (1-3) using ELISA test commercially available, following the manufacturer's protocols (R&D Systems, Minneapolis, MN, USA).

Statistical analysis

All data in text and figures are provided as means ± standard deviation (SD) To compare the three different conditions, we have adopted the One-way ANOVA and Bonferroni adjustment. Statistical analysis was carried out with the statistical software package GraphPad Prism 5.0 (GraphPad Software).

Supplementary Results

Morphologic and Immunophenotypic characterization of UC-MSC

In primary cultures, typical spindle-shaped adherent cells were observed migrating from the UC tissue fragments and initiating the colony formation approximately at day 14 after UC fragments seeding. After removing the UC fragments at day 14, cells at P0 needed approximately 10-days period to gain 60% confluence, while full confluence was observed after 14 days. The UC-MSC clones (P0) were then collected at day 28 and replated for further expansion (P1). Confluence at P1 was observed after 14 days of culture (day 42).
The phenotype of UC cells was analyzed by flow cytometry. The majority of collected UC cells showed a positive expression of the main MSC markers CD73, CD90, and CD105, as well as of CD44 and CD29. Furthermore, they were negative for the typical hematopoietic marker CD34. The data also demonstrated the presence of HLA-ABC proteins and the absence of HLA-DR (data not shown).

Immunofluorescence analysis

Immunofluorescence analysis revealed the presence of scleraxis, collagen type I (Col I), and cell proliferating nuclear antigen (PCNA) marker. Time 0 analysis is shown in the Supplemental figure 1.

Scleraxis

At day 7, a greater presence of scleraxis was observed for samples submitted to PEMF starting from the exposure protocol of 4 hours/day. The intensity of fluorescence (IF) in FGF-2 samples was higher than that of control group. There was a progressive increase in the absolute values of intensity of fluorescence when increasing the daily exposure time to PEMF (2h: m = 15.55, SD = 6.58, 4h: m = 17.07, SD = 5.46, 8h: m = 26.46, SD = 6.82).
At day 14, the results were similar to the previous ones with regard to statistical significance, very similar in the absolute values of intensity on fluorescence (2h: m = 14.61, SD = 3.80, 4h: m = 16.89, SD = 5.78, 8h: 24.63, SD = 6.53).
From 21 days there was a significant increase in the intensity of fluorescence of scleraxis in the samples subjected at least 2 hours of daily PEMF compared to the other conditions. The maximum production of scleraxis, as noted in the chart, was found in samples exposed to 8 hours of exposure (m = 29.77, SD = 11.38).

Collagen Type I

From day 7 samples submitted to any time protocol of PEMF administration showed a significantly higher IF values than that of the two control conditions, with significant differences in the 2 and 8 hour frames (2h: m = 20.99, SD = 6.55, 8h: m = 25.66, SD = 8.31). The only presence of FGF-2 during the culture allowed for a greater production of collagen I than that of the control condition without FGF
At day 14, cells in cultures exposed to PEMF expressed significantly greater amounts of Col I. There was also a gradual increase in the absolute values of IF increasing the daily exposure time (2h: m = 17.93, SD = 7.17, 4h: m = 19.66, SD = 6.23, 8h: m = 31.09, SD = 8.67).
At the end of the three weeks collagen type I IF was significantly greater in the three groups exposed to PEMF than that in the other two conditions of culture. The 8-hour exposure protocol met the significance criteria with a p-value <0.0001 compared to that of unexposed culture. There was no greater collagen I expression in FGF-2 cultures without PEMF than that of the control group without PEMF and FGF.

PCNA

After a week of PEMF exposure the PCNA expression was not significantly higher than that of group with FGF-2 without PEMF, except for the 4 hour protocol (figure 7). In any case, the PEMF exposure or the presence of FGF-2 did not cause a lower expression of PCNA than control group

At day 14, an inversion in the trend of PCNA expression was observed, with a significantly greater IF in the sample cultivated with FGF-2 but not exposed to PEMF compared to those exposed for 4 hours/day. There was no significant difference between 8 hour group and control group.

At the end of the three weeks, in the culture exposed to PEMF, PCNA was expressed in significantly smaller amounts than those of the unexposed cultures. Cultures exposed to PEMF for 4 and 8 hours/day expressed lower PCNA level compared to the FGF-2 group.

Analysis of IL-10 and VEGF levels

The immunoenzymatic test provided data on the presence of two major cytokines with modulating action on the immune and inflammatory response: IL-10 (Interleukin 10) and Vascular Endothelial Growth Factor (VEGF).

Comparison of culture exposed to PEMF and FGF-2

Analysis of IL-10 production revealed linear growth in protocols exposure at 4 and 8 hours, with a peak at day 21 respectively of 67.35 and 51.94 pg/ml. The curves slope increased with the progressive increase of PEMF exposure, with values shift between day 7, 14 and 21

The graph based on changes in VEGF-A values at day 7, 14 and 21 showed a remarkable increase in VEGF values starting from day 14 in the PEMF exposure protocol of 8 hours/day until reaching the maximum value of 974.98 pg/ml recorded at day 21 versus 58.74 pg ml of 2h exposure and 69.23pg/ml of exposure to 4 hours.

Comparison of different culture conditions

Observing graphs for IL-10, we noticed that in all three protocols at day 7 there were no major differences in the concentrations between the three culture conditions. For control culture (-FGF-2, -PEMF), there was a stable value from day 7 to day 14, followed by a fall in day 21 (from 37.88 to 31.40 pg/ml). In culture with FGF-2 not exposed to PEMF, stationary concentrations were found around values of 38 pg/ml. PEMF Exposure protocols with the greatest difference in IL-10 release from tenocytes were those at 4 and 8 hours, day 14, and above all 21 days

With regard to VEGF, concentrations in non-exposed PEMF culture group were lower than those of culture exposed to the three schedule protocols. Cultures with only FGF-2 produced concentrations growing from VEGF starting from the second week, thus passing from 7.57 pg/ml to 9.40 pg/ml at the end of three weeks . The largest deviation in VEGF production between exposed and unexposed specimens was recorded for the 8 hours daily exposure, with values of 3.6 (day 7) to 21 (day 21) times higher than culture with the only FGF-2

Cell viability analysis

The Annexin V and Propidium Iodide are able to selectively mark cells, then detected through the use of flow cytometer, in apoptosis or necrosis. The charts allow for observing the percentage of live cells (double negative, in the lower left quadrant), necrotic (negative to annexin and positive to PI, upper left quadrant), late apoptosis (double positive, upper

right quadrant) and early apoptosis (positive to annexin and negative to PI, lower right quadrant).
The cell mortality data at day 7 of PEMF exposure showed 95.23% survival rate for 2h/day protocol, 80.83% at 4h, 90.17% at 8h, with the highest percentage of necrosis and apoptosis (PI positive cells or PI and annexin) found in 4h protocol
At day 14, there was a decrease in the percentages of surviving cells in all the three cultures, in particular in 8h protocol, in which, besides a decrease of about 17.5% of live cells, there was a percentage of 14,87% cells in early apoptosis. The lowest percentage of cell survival was reported in 4h exposure protocol (65.86%) with 25,79% positive double cells.
At the final time point at three weeks (day 21), we found a further decrease in cell survival in the 2 and 4 hours protocols (respectively 19% and 17%), consensually with the negative trend already recorded in the previous analyses. In the 8-hour culture, however, there was a very high percentage of surviving cells (91.95%) compared to day 14 (72.67%)

Supplemental Figure 1

Scleraxis, Type I Collagen and PCNA expression at time 0
Immunofluorescent analysis for Scleraxis, Type I Collagen and PCNA expression at time 0 of cell culture

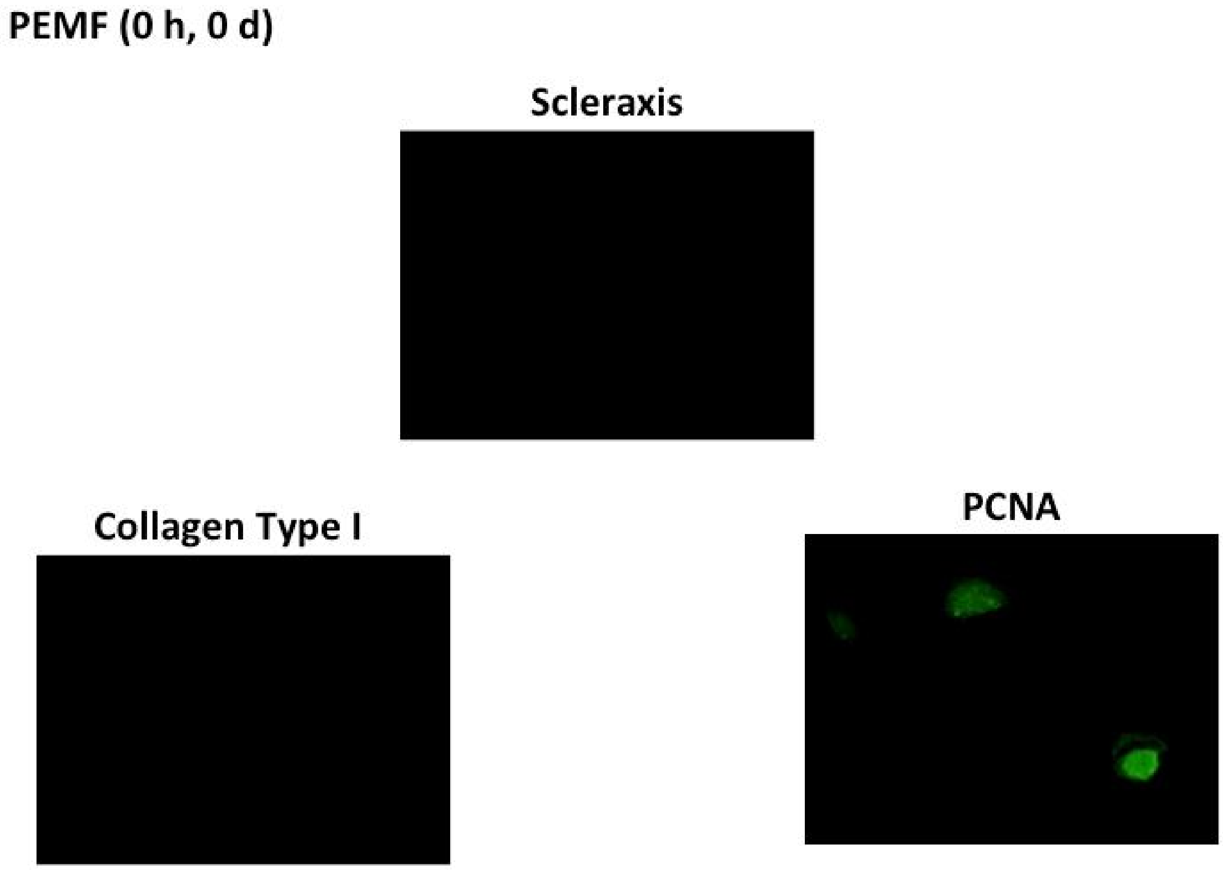

Supplement: Supplementary Materials — Supplementary Results: this section clarifies the details of the numerical values of the different parameters depicted in the figures and previously described in Results of the main manuscript. [file 9048237.f1.pdf]
